# Genomic and non-genomic pathways are both crucial for peak induction of neurite outgrowth by retinoids

**DOI:** 10.1186/s12964-019-0352-4

**Published:** 2019-05-02

**Authors:** Thabat Khatib, Pietro Marini, Sudheer Nunna, David R. Chisholm, Andrew Whiting, Christopher Redfern, Iain R. Greig, Peter McCaffery

**Affiliations:** 1 School of Medicine, Medical Sciences and Nutrition, Institute of Medical Sciences, University of Aberdeen, Foresterhill, Aberdeen, AB25 2ZD Scotland, UK; 2 Department of Chemistry, Durham University, South Road, Durham, DH1 3LE UK; 3 Northern Institute for Cancer Research, Medical School, Newcastle University, Newcastle upon Tyne, NE2 4HH UK

**Keywords:** Retinoic acid, Vitamin a, Erk1/2, Neurite outgrowth, RAR, Transcription, Non-genomic

## Abstract

Retinoic acid (RA) is the active metabolite of vitamin A and essential for many physiological processes, particularly the induction of cell differentiation. In addition to regulating genomic transcriptional activity via RA receptors (RARs) and retinoid X receptors (RXRs), non-genomic mechanisms of RA have been described, including the regulation of ERK1/2 kinase phosphorylation, but are poorly characterised. In this study, we test the hypothesis that genomic and non-genomic mechanisms of RA are regulated independently with respect to the involvement of ligand-dependent RA receptors. A panel of 28 retinoids (compounds with vitamin A-like activity) showed a marked disparity in genomic (gene expression) versus non-genomic (ERK1/2 phosphorylation) assays. These results demonstrate that the capacity of a compound to activate gene transcription does not necessarily correlate with its ability to regulate a non-genomic activity such as ERK 1/2 phosphorylation. Furthermore, a neurite outgrowth assay indicated that retinoids that could only induce either genomic, or non-genomic activities, were not strong promoters of neurite outgrowth, and that activities with respect to both transcriptional regulation and ERK1/2 phosphorylation produced maximum neurite outgrowth. These results suggest that the development of effective retinoids for clinical use will depend on the selection of compounds which have maximal activity in non-genomic as well as genomic assays.

**Electronic supplementary material:**

The online version of this article (10.1186/s12964-019-0352-4) contains supplementary material, which is available to authorized users.

## Background

Retinoids are a family of natural or synthetic compounds that are analogues of vitamin A and its derivatives [1]. They are essential for numerous cellular activities including growth, proliferation and differentiation of a variety of tissues, and are necessary for central nervous system development, neuroplasticity and neurite outgrowth [2]. Most actions of retinoids are mediated by retinoic acid (RA), which is derived from retinol in a two-step enzymatic process via a retinaldehyde intermediate [3].

Retinoic acid (RA) has been described as a neurotrophic factor that binds to nuclear receptors (RAR and RXR), inducible ligand-activated transcription factors which regulate multiple physiological mechanisms at a genomic level [4, 5]. In addition to this ‘classical’ mechanism, RA can exert non-genomic effects, mediated either by the RARs or independent of them [6–8]. Several studies have demonstrated that RA can activate kinases, such as ERK1/2 [9–13], which may play a role in cytoskeletal rearrangement and neurite outgrowth [14] in a number of cell types. The RA signalling pathway also regulates neurite outgrowth [15], a property demonstrated in several in vitro models including neuroblastoma cell lines and primary neuron cultures [16–21]. Whether this is a result of genomic or non-genomic effects, or a combination of such mechanisms, is unknown.

A large number of synthetic retinoids have been generated as agonists of the RA nuclear receptors, but, in contrast to the large number of published studies on their regulation of gene expression, very few studies have characterised the non-genomic action of retinoids in relation to their properties as RAR ligands. In this study, we test the hypothesis that genomic and non-genomic activities of retinoids are regulated independently using a diverse range of commercially-available, and novel RAR and RXR ligands for several of the different classes of RA receptors (RARα/β/γ, RXRα/β/γ) or ligands for RBP4. The properties of these ligands were compared with respect to activity in genomic (gene expression) assays and a non-genomic (ERK1/2 phosphorylation) assay, and their ability to induce neurite outgrowth.

## Methods

### Retinoid solutions

**Fig. 1:**
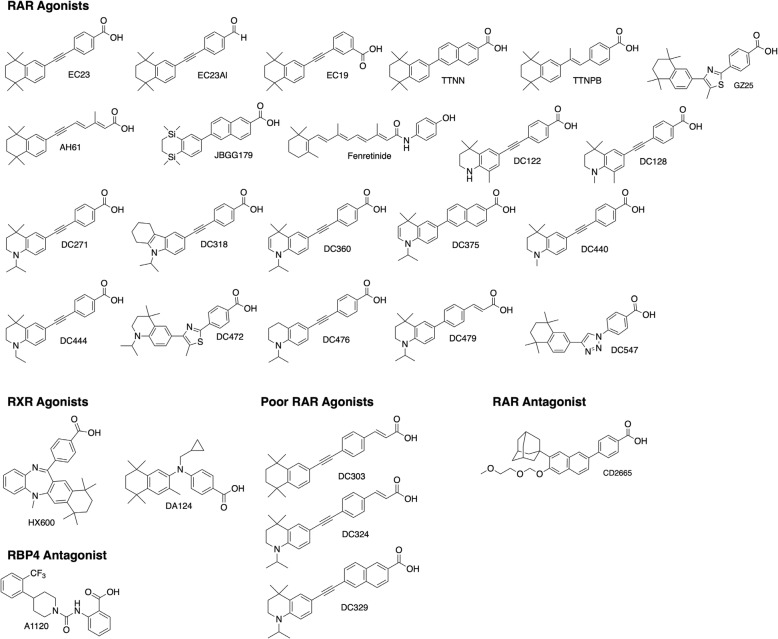
Retinoids used in the study grouped into those that are RAR agonists, RXR agonists, RAR antagonists, poor RAR agonists and antagonists for RBP4

All-*trans*-RA (ATRA) (Sigma-Aldrich) was dissolved at a concentration of 0.1 M in dimethyl sulfoxide (DMSO) under red light in a dark room, aliquoted into 0.6 ml microtubes stored under nitrogen at − 70 °C, and protected from light. Synthetic retinoids were dissolved in DMSO as 0.01 M stock solutions and stored at − 20 °C, protected from light. A1120 and TTNPB were purchased from Sigma-Aldrich, CD2665 was purchased from Tocris; HX600 and DA124 were obtained from Dr. Kagechika (Tokyo) while fenretinide was obtained from Dr. Nimesh Mody (University of Aberdeen). Other synthetic retinoids and non-retinoid homologues were designed and synthesized as described previously [22–28]. The molecular structures of the RAR and RXR ligands used are shown in Fig. 1. The majority of the compounds are effective activators of RARs, except for A1120 (a RBP4 ligand), HX600 and DA124 (RXR agonists), CD2665 (RAR β/γ antagonist) and DC324, DC329 and DC303, which exhibit extended structures compared to their shorter analogues (DC271, DC375 and EC23, respectively) that significantly reduce their binding affinities for the RARs.

### Cell culture

Sil-15 cells [29] were used in the X-Gal based RA reporter assay, described below, to measure the transcriptional activity of ATRA and other RAR and RXR ligands. This cell line was derived [29] from F9 teratocarcinoma cells transfected with a plasmid containing the LacZ gene under the control of a promoter linked to the RARβ RARE [30, 31]. Sil-15 cells were cultured at 37 °C with 5% CO_2_ in T-75 flasks in Dulbecco’s Modified Eagle’s Medium (DMEM; Thermo Fisher Scientific) containing 10% fetal calf serum (FCS; Thermo Fisher Scientific), 0.8 mg/ml G418 disulfate salt (Geneticin, Sigma-Aldrich) to maintain selection for the LacZ plasmid; cells were passaged twice a week using 0.05% Trypsin-EDTA.

SH-SY5Y cells [32] were used in the neurite outgrowth, ERK phosphorylation and RA genomic activity assays. The cells were treated with varying concentration of retinoids for each assay depending on the results of previously performed pilot studies. These cells are a third subclone of the SK-N-SH neuroblastoma cell line [33]. SH-SY5Y cells were grown in DMEM containing 10% FCS at 37 °C with 5% CO_2_. The medium was changed three times a week and the cells were passaged at 70% confluence using 0.05% trypsin-EDTA. The passage number used for each experiment was no higher than 30.

### X-gal based RA reporter assay

Sil-15 cells were used to detect and quantify the transcriptional activity of retinoids added to the medium by monitoring β-galactosidase activity produced by the reporter cells. 96-well plates were coated with 0.1% gelatin for at least 2 h at 37 °C, washed twice with PBS, wrapped with parafilm and stored at 4 °C until use. Sil-15 cells were removed from stock culture flasks by trypsinization, counted and plated at 100,000 cells per well in the pre-coated 96-well plates. After attachment overnight in DMEM containing 10% FCS, the medium was replaced with fresh DMEM/10% FCS and serial dilutions of retinoid ligands, prepared in DMEM containing 10% FCS, were added at concentrations from 10^− 6^ M to 10^− 14^ M. The plates were incubated overnight at 37 °C / 5% CO_2_. All concentrations for the ATRA standard curve and the other retinoid ligands were tested in triplicate. The next day, the assay plates were washed twice with PBS, fixed with 100 μl per well of 1% glutaraldehyde and 1 mM MgCl_2_ in PBS for 15 min, washed twice with PBS and β-galactosidase activity detected by adding to each well 100 μl of freshly-prepared 0.2% X-Gal in 1 mM MgCl_2_, 3.3 mM potassium ferricyanide and 3.3 mM potassium ferrocyanide in PBS. Plates were incubated for 6 h at 37 °C in 5% CO_2_ and colour change at 650 nm measured on an Emax Precision Microplate Reader (Molecular Devices).

### Gene expression analysis

Total RNA was extracted from SH-SY5Y cells treated with 10 nM retinoid ligands for 24 h using a Qiagen RNeasy mini kit according to the manufacturer’s protocol. An extra DNAse digestion step was carried out during RNA purification to remove DNA contamination. cDNA was synthesized from 250 ng total RNA using High qScript cDNA Synthesis master mix. qPCR reactions using PerfeCTa SYBR Green SuperMix were performed on a Roche LightCycler 480 and analyzed using LightCycler 480 1.5 software. Primers spanning exon-exon junctions were designed using Primer-BLAST [34] for *CYP26A1* (CACCGTACGGGTGATGGGCG, GCTGGCCAGTGGACCGACAC), *RARβ* (GCTTAATCTGTGGAGACCGCCAGG, TGTGAGGCTTGCTGGGTCGT), and *GAPDH* (TCTTTTGCGTCGCCAGCCGA, AGTTAAAAGCAGCCCTGGTGACCA) genes. Standard curves and blank controls were run for all sets of primers tested. A housekeeping gene was chosen for normalization by prescreening a range of genes with experimental samples and choosing the one showing least variability between samples, which in this case was *GAPDH*.

### ERK1/2 phosphorylation screening using the AlphaLISA® SureFire® Ultra assay

The ability of ATRA and other RAR and RXR ligands to phosphorylate ERK1/2 was determined in SH-SY5Y cells using the AlphaLISA® SureFire® Ultra ERK1/2 kit (PerkinElmer). In this assay, SH-SY5Y cells (100,000 cells/well) were plated in 96-well plates and serum-starved in DMEM for 24 h. Retinoids were tested at concentrations from 10^− 5^ M to 10^− 11^ M and at a final concentration of 0.1% DMSO in the medium. SH-SY5Y cells were assayed in serum-free DMEM and stimulated for 30 min (determined from a time course experiment) at 37 °C. At the end of the assay, the medium was removed, and cells were lysed with 50 μl of freshly prepared 1X lysis buffer supplied in the kit. The 96-well plate was agitated on an orbital shaker SO1 (Stuart Scientific) at approximately 350 rpm for 10 min at room temperature. In the meantime, the activation buffer was diluted 25-fold in the reaction buffers. Under green light in a dark room the acceptor beads were diluted 50-fold in the freshly prepared reaction mix while the donor beads were diluted 50-fold in dilution buffer to obtain two final reaction mixtures. 10 μl of cell lysate was then transferred to the wells of a 384-well white Proxiplates plate (PerkinElmer) and 5 μl of each prepared acceptor and donor reaction mixtures was added above the wells while still under green light in the dark room. Plates were next wrapped with aluminium foil and incubated at room temperature for at least 3 h and read with the Envision system (PerkinElmer Life Sciences) using AlphaScreen® settings.

### Neurite outgrowth assay

16 mm glass coverslips were acid treated in a mixture of 69% nitric acid and 37% hydrochloric acid in a 2:1 ratio for 2 h. The coverslips were then washed extensively with MilliQ deionised water until the pH of the water reached 5.5–6. Until needed, the coverslips were stored in a jar containing 70% ethanol. The coverslips were put on a 70% ethanol-soaked tissue paper under the cell culture hood to dry. The coverslips were then placed in the wells of 12-well plates. After that they were coated by addition of 1 ml of poly-L-lysine (PLL) solution above each coverslip in the well. The final concentration of PLL solution used for coating was 0.002% and the incubation time was 2 h at 37 °C. Two washes with sterile PBS followed and afterwards the coverslips were air dried under sterile conditions.

SY-SY5Y cells were removed from stock flasks by trypsinization, counted and plated at 10,000 cells/well in 12-well plates containing acid-treated/PLL-coated coverslips. The plates were maintained at 37 °C in a humid atmosphere containing 5% CO_2_ for 24 h. Then, each retinoid was added to the medium and tested at two different concentrations, 10 μM and 10 nM, with a final DMSO concentration of 0.01% or 0.0001%, respectively. The plates incubated for 5 days at 5% CO_2_ / 37 °C. All retinoid concentrations were tested in triplicate.

After retinoid treatment, SH-SY5Y cells on coverslips were washed twice in PBS, fixed in 4% paraformaldehyde (PFA) for 20 min at room temperature, followed by two washes with PBS. Coverslips were stored at 4 °C in PBS until stained. For immunocytochemical staining of neurites, cells on coverslips were washed three times in PBS, and incubated in blocking solution (10% donkey serum and 0.1% Triton X-100 in PBS) for 1 h at room temperature. Cells were then labelled by incubation overnight at 4 °C with β-III tubulin primary antibody (Sigma-Aldrich) diluted 1:1000 in blocking buffer, washed three times with PBS containing 0.1% Triton X-100 solution (PBST) before incubation with anti-mouse monoclonal secondary antibody (1:300 in PBST; Jackson Immunoresearch) for 2 h at room temperature. Finally, after three washes in PBST and a final wash in PBS, the coverslips were mounted on slides and stored at 4 °C.

ImageJ software with the NeuronJ plugin was used to quantify neurite outgrowth on stained cells. For each experiment, 10 different randomly selected images were taken from each cover slip using a Nikon Eclipse E400 fluorescence microscope. Each image was converted into an 8-bit image (necessary for the NeuronJ plugin) and optimised with the brightness and contrast tool in GIMP (GNU Image Manipulation Program). For each image, individual traces were drawn for each clearly-identifiable neurite using the tracing tool in the NeuronJ plugin. Neurite length was measured in pixels and transformed into the corresponding length in μm depending on the magnification used. The average neurite length for each image was calculated by dividing total neurite length by the total number of neurites per image. Ten images per cover slip were measured and the mean calculated for the coverslip overall. Coverslips were in triplicate for each retinoid and concentration.

### Cell number assay

SH-SY5Y cells were plated on acid-treated/PLL-coated coverslips, treated with retinoids as described previously in the neurite outgrowth assay section, 4% PFA fixed and stained with bisbenzimide dye (1 mg/ml). For each experiment, 10 different randomly selected images were taken from each cover slip using a Nikon Eclipse E400 fluorescence microscope. The number of cells were then counted in each image manually, identifying each cell from its bisbenzimide-labelled nucleus. Ten images per cover slip were measured and the mean calculated for the coverslip overall. Coverslips were in triplicate for each retinoid and concentration.

### Statistical analysis

All data are presented as mean ± SEM of three independent experiments with biological triplicates. Statistical analyses were performed in Microsoft Office Excel 2017, GraphPad Prism 7.0c version (Prism, GraphPad Software, San Diego, CA) and R version 3.3.1 (R Core Team, 2016) [35]. Gene expression and Neurite Outgrowth data were analysed by Student’s t-test or one-way ANOVA with Newman-Keuls multiple comparison test as appropriate; *P* value < 0.05 was considered statistically significant. * *P* < 0.05, ** *P* < 0.01, *** *P* < 0.001, **** *P* < 0.0001. Data for X-Gal and ERK1/2 phosphorylation were analysed using sigmoidal dose-response analysis of log (agonist) versus response curve (stimulation). Non-parametric (Spearman) and parametric (Pearson) correlation analyses were used to compare cell responses, and asymptotic Wilcoxon-Mann-Whitney tests for non-parametric two-way comparisons of neurite length and cell number, where appropriate. Non-parametric correlation analyses were used for comparisons involving transcriptional data so that outliers could be included without bias. The results of analyses were presented as E_max_ and EC_50_ with 95% confidence interval limits (95% CI).

## Results

### Genomic activity of retinoids

**Fig. 2:**
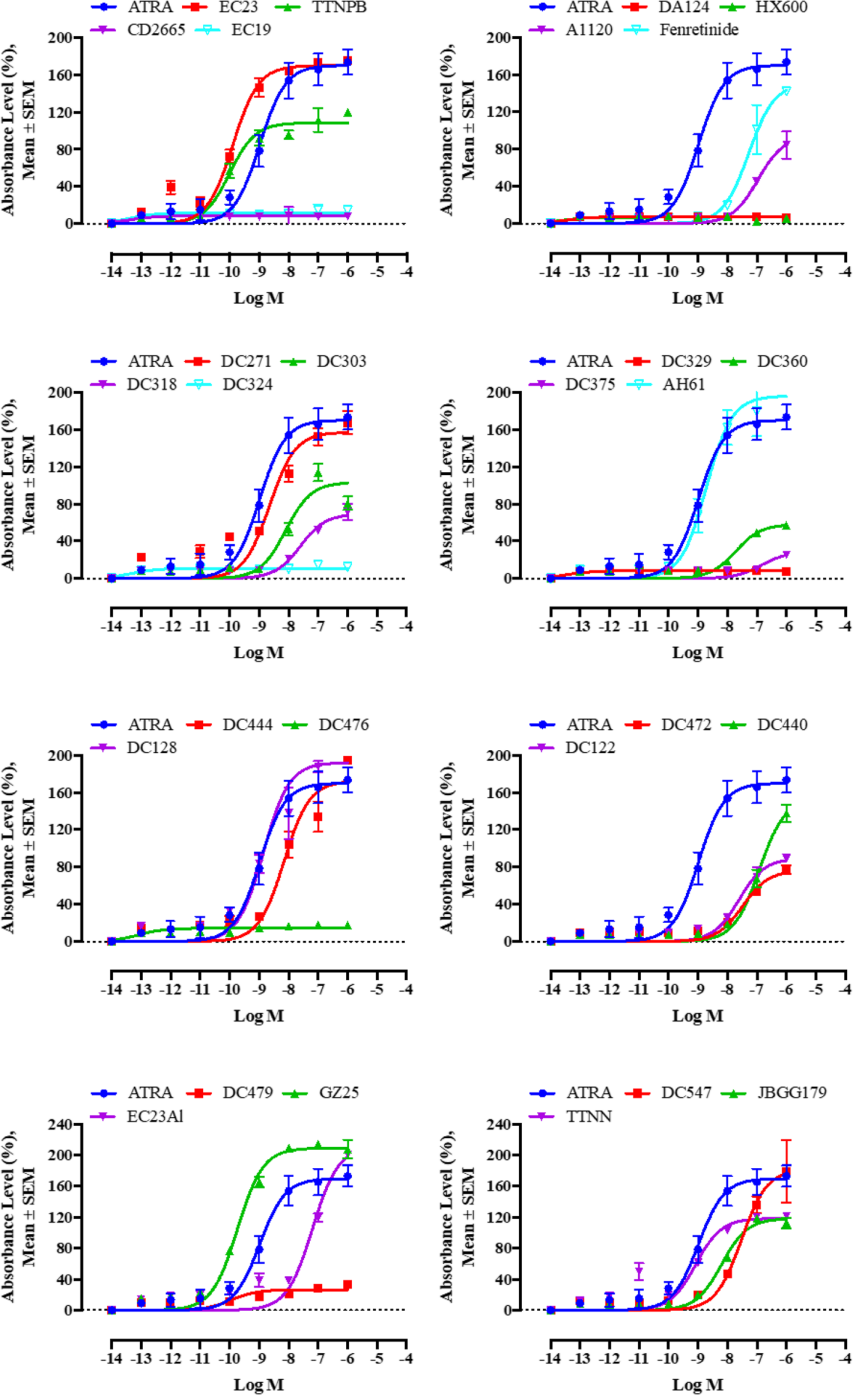
The concentration-response graph for log (agonist) vs. sigmoidal dose-response in evaluating ATRA versus retinoids in inducing genomic response of Sil-15 reporter cells. Absorbance values of different retinoid doses were measured at 650 nm and analysed using sigmoidal dose-response curves. Shown are average absorbance of three independent experiments. Error bars indicate standard error of the mean (SEM). Statistically significant differences are indicated by non-overlapping 95% CI

To quantify genomic (transcriptional regulation) activity, the Sil-15 RA reporter cell line, in which X-gal expression is driven by a RARβ RARE, was used to assay retinoids at concentrations ranging from 10^− 6^ M to 10^− 14^ M. In these assays (Fig. 2), the EC_50_ value represents the half-maximal effective concentration (potency) and is related to the affinity for the receptor; the maximum stimulation, defined by the E_max_ value, is a measure of the compound efficacy. Of the 28 retinoids tested, nine (HX600, DA124, EC19, CD2665, DC375, DC324, DC329, DC476 and DC479) did not have any genomic activity compared to ATRA. However, 19 compounds were effective in inducing a genomic response. Three retinoids, EC23, TTNPB and GZ25, had significantly lower EC_50_ than ATRA and TTNN, DC128, AH61, and DC271 had similar potencies to ATRA; the other retinoids had significantly higher EC_50_ values than ATRA (Fig. 2).

In addition, the compounds varied in their efficacy (E_max_). Not all compounds with higher potency compared to ATRA also exhibited higher efficacy, and vice versa. For example DC271 and TTNPB had lower E_max_ values, even though their EC_50_ values were lower or the same as ATRA. EC23Al (the aldehyde of EC23) showed a significantly higher E_max_ while the EC_50_ was greater compared to ATRA. Moreover, GZ25 exhibited a significantly higher E_max_ compared to ATRA, and also showed greater potency than ATRA. The potency and efficacy of each ligand along with the 95% confidence intervals (CI) in inducing a genomic activity are summarised in Additional file 1 :Table S1.

**Fig. 3:**
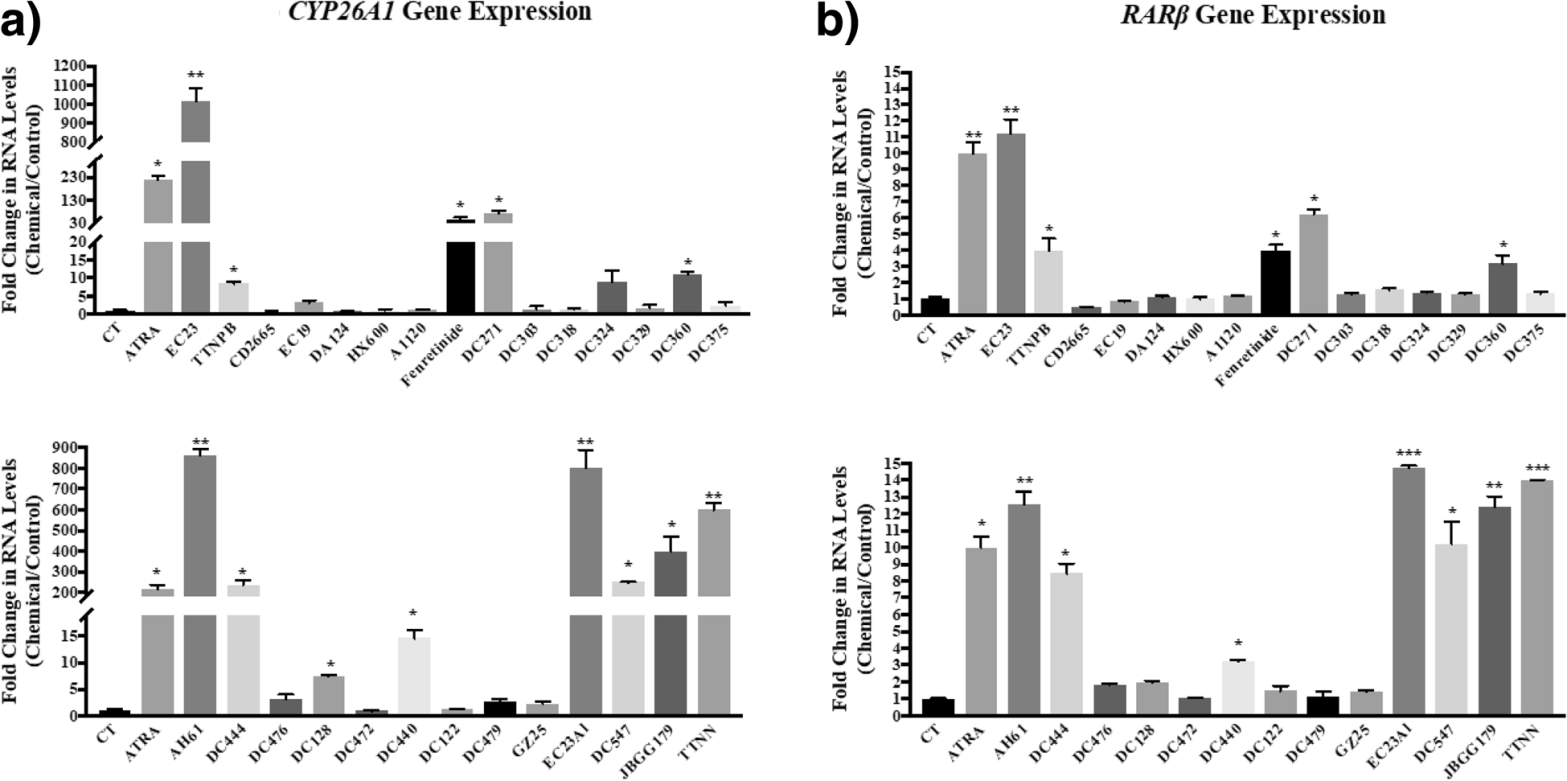
Analysis of the effects of 10 nM retinoid treatment on SH-SY5Y cells according to *CYP26A1* and *RARβ* RNA levels by RT-qPCR. SH-SY5Y cells were treated with 10 nM retinoids for 24 h. RNA was isolated and **a**) *CYP26A1* or **b**) *RARβ* RNA analysed by qPCR. *CYP26A1* and *RARβ* RNA levels were standardised with respect to the *GAPDH* RNA control and compared to levels in control untreated cells (CT) which were set at 1. Shown are mean values of three independent experiments analysed in triplicate. Error bars are SEM (* *p* < 0.05; ** *p* < 0.01, one-way ANOVA with Newman-Keuls multiple comparison test). Several of the tested RAR and RXR ligands increased the expression levels of *CYP26A1* and *RARβ* genes significantly above control indicative of transcriptional activity, however, few were more potent than ATRA

To confirm the activity of these retinoids on endogenous transcriptional regulation in the SH-SY5Y cell line used for neurite outgrowth and non-genomic activity assays, two genes with well-defined RAREs in their promoters were investigated: *RARβ* and *CYP26A1* [30, 36–38]. In these assays, the fold change in *RARβ* and *CYP26A1* gene expression in SH-SY5Y cells treated with 10 nM concentrations of RAR and RXR ligands were compared to a DMSO control (Fig. 3). EC23, AH61, EC23Al, TTNN and JBGG179 all had greater potency than ATRA at inducing *CYP26A1* and *RARβ*. DC444 induced *CYP26A1* and *RARβ* gene expression to almost the same extent as ATRA. Fenretinide, DC440 and DC271 induced *CYP26A1* and *RARβ* but were weaker than ATRA, while DC360 and DC476 only induced *RARβ.* The other RAR and RXR ligands did not activate *CYP26A1* or *RARβ.* These gene expression results were generally comparable to the results obtained with the X-Gal RA based reporter assay; a key result is that GZ25 and EC23 were stronger in their genomic activity compared to ATRA at low concentrations in both assays.

### Non-genomic activity (ERK1/2 phosphorylation) induced by retinoids in SH-SY5Y cells

**Fig. 4:**
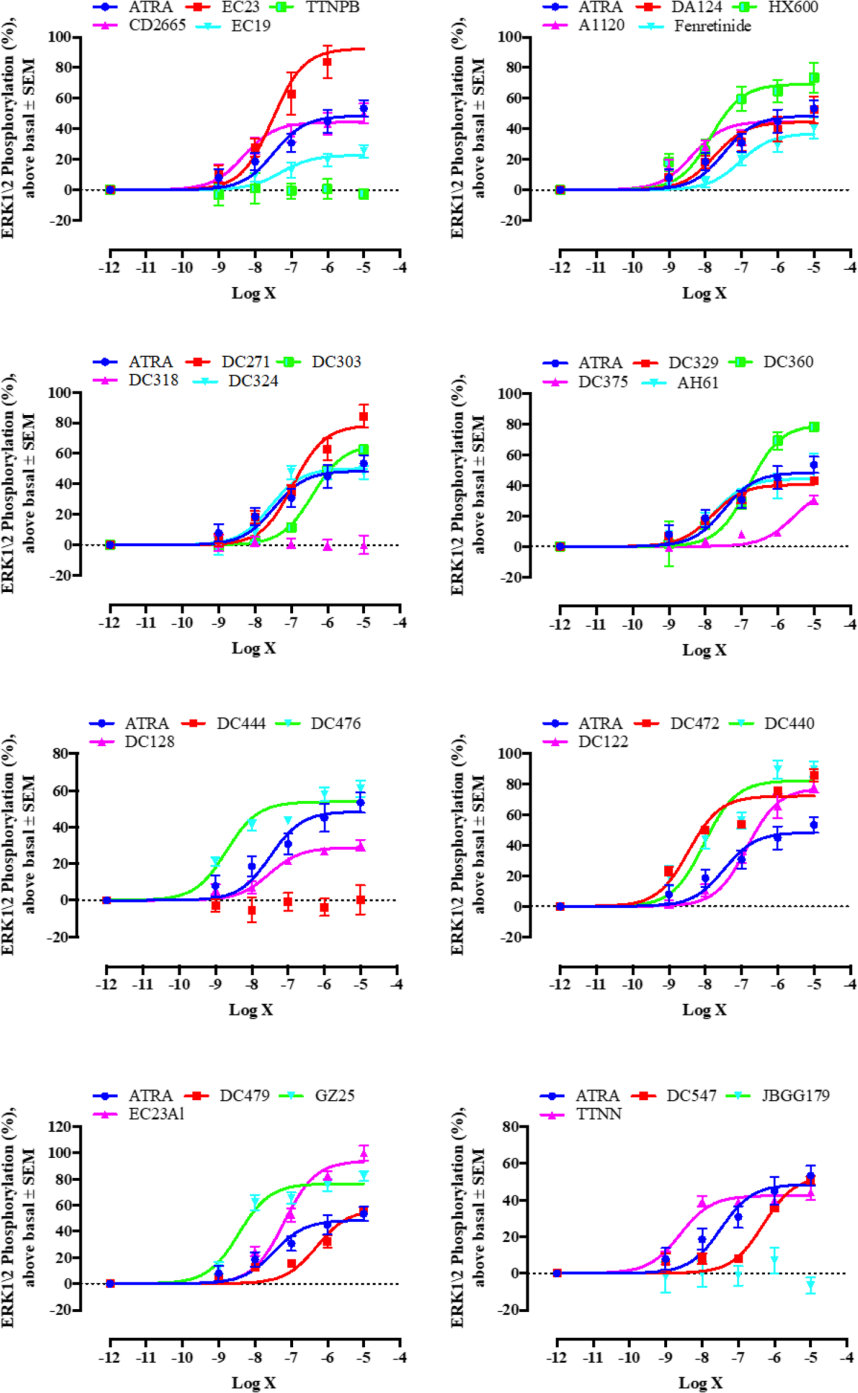
Sigmoidal concentration-response graphs for induction of ERK1/2 phosphorylation in SH-SY5Y cells. Absorbance values of different retinoid doses were measured at 570 nm. The average absorbance in three independent experiments are shown. Error bars indicate SEM. There was a statistical difference in the potency (EC_50_) between ATRA and (A1120, CD2665, HX600, GZ25, DC329, DC440, DC472, DC476, TTNN), and the efficacy (E_max_) between ATRA and (EC23, EC23Al, HX600, GZ25, DC122, DC271, DC303, DC360, DC440, DC472, DC476, DC479) calculated by the non-overlapping 95% CI

SH-SY5Y cells have been reported to respond in a non-genomic manner to the nuclear receptor ligand, ATRA, with an increase in ERK1/2 activity [39–41]. Therefore, changes in ERK1/2 activity, detected as ERK1/2 phosphorylation, in response to retinoids were compared to ATRA as a positive control (Fig. 4). In these experiments we could confirm that ATRA was a strong activator of ERK1/2 kinase phosphorylation [40, 42, 43]. Of the other retinoids, nine (A1120, CD2665, HX600, GZ25, DC329, DC440, DC472, DC476 and TTNN) induced ERK1/2 phosphorylation with an EC_50_ that was significantly lower than ATRA. Five ligands, AH61, EC23, DA124, DC128 and DC324, had EC_50_ values for ERK1/2 phosphorylation similar to ATRA. The remaining retinoids were less potent compared to ATRA (Fig. 4).

With respect to efficacy, 13 of the RAR and RXR ligands (EC23, EC23Al, HX600, GZ25, DC122, DC271, DC303, DC360, DC440, DC472, DC476, DC479 and DC547) had E_max_ values significantly higher than ATRA. Six retinoids, AH61, A1120, CD2665, DA124, DC324 and TTNN, had similar efficacies to ATRA. The efficacy of the rest of the retinoids was significantly lower than ATRA. The potency and efficacy of each ligand along with the 95% confidence intervals (CI) in inducing ERK1/2 phosphorylation are summarised in Additional file 1 :Table 1.

### Neurite outgrowth and number of SH-SY5Y cells increased by retinoids

**Fig. 5:**
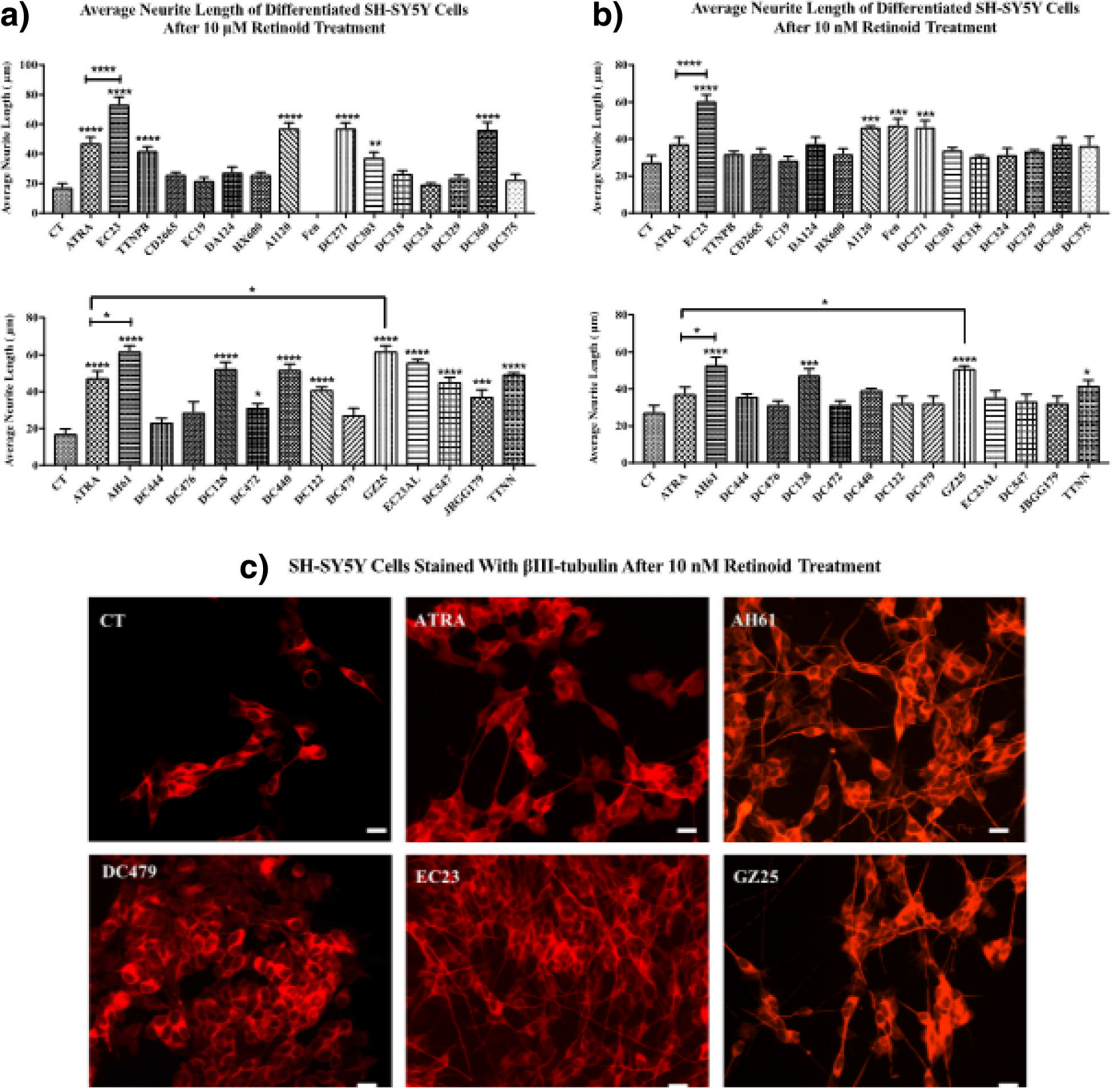
Neurite outgrowth of SH-SY5Y cells treated with retinoids. SH-SY5Y cells were treated with the retinoids or DMSO CT treatment for 5 days, and then neuronal differentiation was evaluated by immunofluorescence and morphometric analysis, using βIII-tubulin antibody. The average length of neurites extending from SH-SY5Y cells after **a**) 10 μM and **b**) 10 nM compound treatment was measured and compared with the control and ATRA. Shown are mean values of three independent experiments. The average neurite length was calculated by dividing the total neurite length by the total number of neurites in each micrograph. Error bars indicate SEM (* *p* < 0.05; *** *p* < 0.001; **** *p* < 0.0001 one-way ANOVA with Newman-Keuls multiple comparison test). **c**) Representative micrographs of SH-SY5Y cells stained with βIII-tubulin antibody after 10 nM retinoid treatment for 5 days (scale bar = 18 μm)

**Fig. 6:**
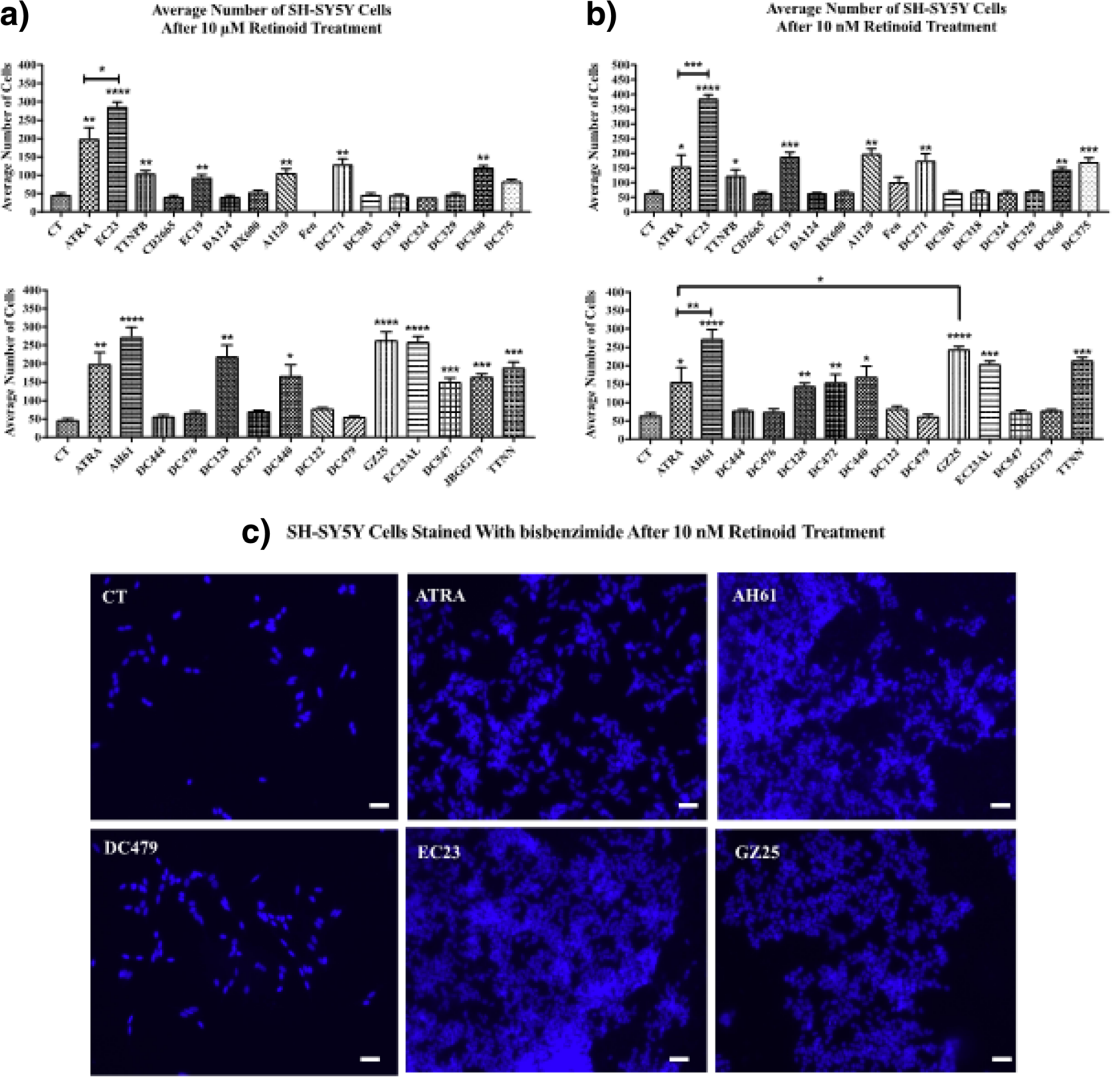
Increase in number of SH-SY5Y cells treated with retinoids. SH-SY5Y cells were treated with the retinoids or DMSO CT treatment for 5 days, and then number of cells evaluated by staining with bisbenzimide dye and counting the cells. The average number of SH-SY5Y cells after **a**) 10 μM and **b**) 10 nM compound treatment was measured and compared with the control and ATRA. Shown are mean values of three independent experiments. Error bars indicate SEM (* *p* < 0.05; ** *p* < 0.01; *** *p* < 0.001; **** *p* < 0.0001 one-way ANOVA with Newman-Keuls multiple comparison test). **c**) Representative micrographs of SH-SY5Y cells stained with bisbenzimide dye after 10 nM retinoid treatment for 5 days (scale bar = 18 μm)

The SH-SY5Y cell line can be induced by ATRA towards a neuronal phenotype and is used as a model to study neuronal differentiation and neurite outgrowth [44]. In addition, ATRA stimulates an increase in cell number in SH-SY5Y cell cultures [9]. Each retinoid was tested at two different concentrations, 10 μM, as an assay of relative efficacy, and 10 nM as a measure of relative potency (Figs. 5 and 6).

As in previous studies, ATRA at 10 μM induced neurite outgrowth of SH-SY5Y cells [45]. EC23, AH61 and GZ25 induced neurite outgrowth of SH-SY5Y cells at the two tested concentrations, and with substantially higher relative potency compared to ATRA. Fenretinide induced death of almost all the SH-SY5Y cells at 10 μM concentration; SH-SY5Y cells have a particular sensitivity to fenretinide which induces apoptosis in a concentration dependent manner [46–48]. Conversely, fenretinide significantly induced neurite outgrowth at 10 nM concentration. A1120 and DC271, DC128 and TTNN also induced neurite outgrowth of the SH-SY5Y cells at the two tested concentrations. TTNPB, DC360, DC303, DC122, DC440, EC23Al and DC547 induced neurite outgrowth at 10 μM concentration only. HX600, DA124, DC318, CD2665, DC375, DC324, DC329, DC476, JBGG179, DC479 and EC19 did not have visible effects on neurite outgrowth in the SH-SY5Y cells at either concentration. The fold increase in neurite outgrowth induced by each ligand is summarised in Additional file 1: Table S1. The number of neurites per cell was also determined but this did not change significantly between retinoid treatment (data not shown).

Cell number was measured in SH-SY5Y cultures after each retinoid treatment (Fig. 6). ATRA, EC23, AH61, GZ25, TTNPB, EC19, A1120, DC271, DC360, DC128, DC440, EC23AL and TTNN increased SH-SY5Y cell number significantly at the two tested concentrations. EC23, AH61 and GZ25 increased the number of cells at 10 nM concentration with significantly higher relative potency compared to ATRA. DC375 and DC472 increased SH-SY5Y cells significantly at 10 nM concentration only. The rest did not have significant effects on the number of SH-SY5Y cells at either concentration.

Overall, there was a significant positive correlation between the increase in neurite length and cell number induced by the retinoids (correlation coefficient, Pearson, 0.77; *P* < 0.00001), while there was no significant correlation between ERK1/2 activity (E_max_) and transcriptional activation activity (E_max_) of the retinoids tested (*n* = 28, Spearman r = 0.2, *P* = 0.29). This suggests that the genomic and non-genomic properties of the retinoids tested are independent.

### Relationship of ERK1/2 kinase activity to neurite outgrowth and cell number

For retinoids that increased neurite length there was no significant correlation between ERK activity (Efficacy, E_max_) and neurite length (correlation coefficient, Pearson, 0.28; *P* = 0.3); conversely, there was a significant positive correlation between neurite length and transcriptional activity (E_max_, correlation coefficient, Spearman, 0.6, *P* = 0.014). This suggests a proportional relationship between genomic properties of retinoids and increasing neurite length but a lack of such proportional relationship with respect to ERK1/2 activity as a non-genomic effect. For cell number, using all data (including retinoids that did not increase neurite length), there was no significant correlation with ERK activity (correlation coefficient, Pearson, 0.315; *P* = 0.1), but, as with neurite length, there was a significant correlation between cell number and transcriptional activity (correlation coefficient, Spearman, 0.745, *P* < 0.0001), again suggesting that the genomic effects of retinoids are important determinants of cell responses. Plots showing correlation analysis are illustrated in Additional file 2: Figure S1.

For retinoids that increased neurite length, significantly shorter neurites were induced by retinoids (*n* = 4) that had no detectable ERK1/2 activity compared to retinoids (*n* = 16) that had activity in both the ERK1/2 and transcriptional assays (Asymptotic Wilcoxon-Mann-Whitney Test; *P* = 0.012). However, this was not the case for increases in cell number (Asymptotic Wilcoxon-Mann-Whitney Test; *P* = 0.11).

**Fig. 7:**
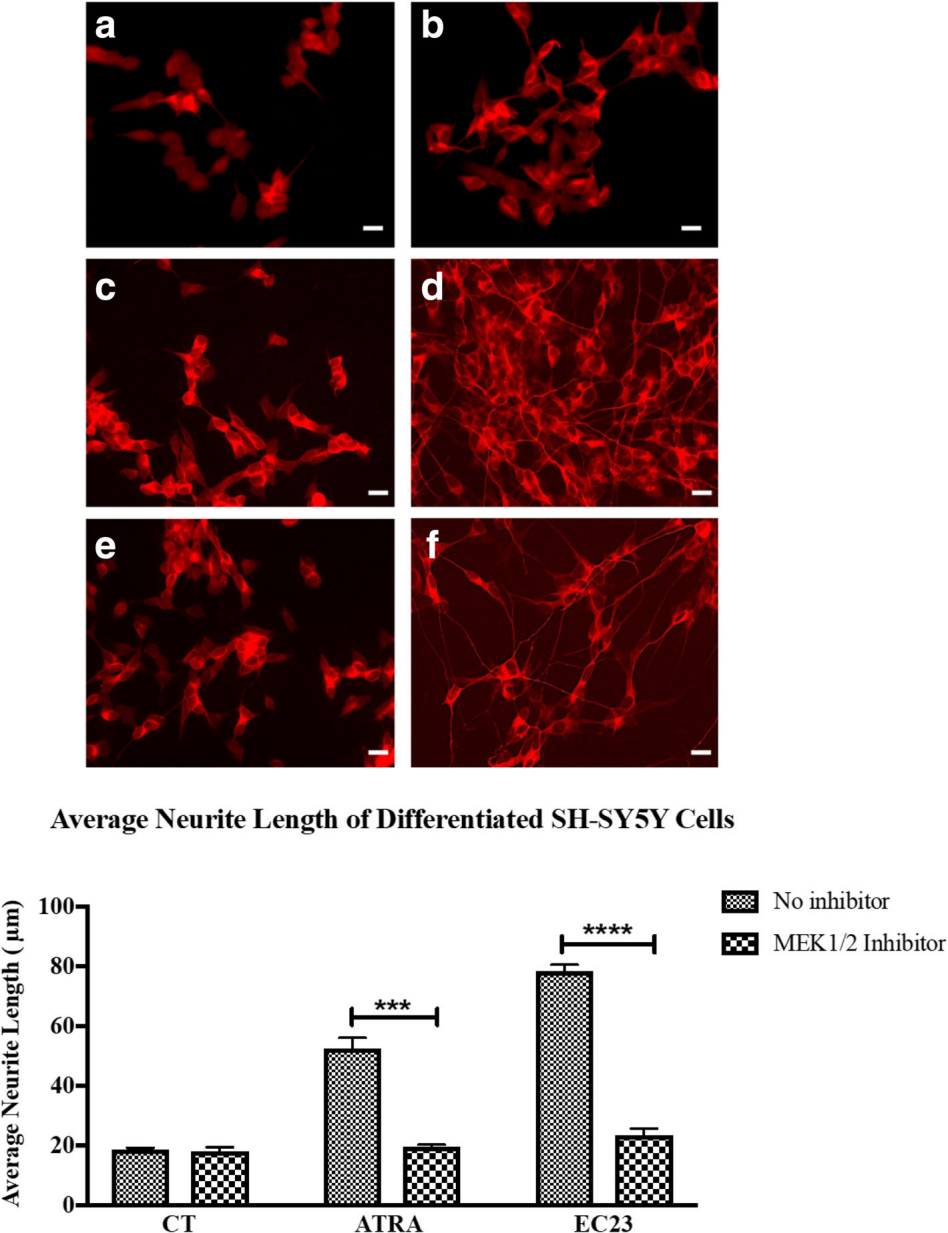
MEK1/2 kinase involvement in neurite outgrowth. SH-SY5Y cells were treated with ATRA and EC23 with/without U0126 MEK1/2 inhibitor for 5 days, and then neuronal differentiation was evaluated by immunofluorescence and morphometric analysis using βIII tubulin antibody. Neurite outgrowth was significantly reduced after applying U0126. Typical micrographs are shown in **a**) DMSO CT, **b**) MEK1/2 inhibitor, **c**) 10 μM of ATRA and MEK1/2 inhibitor, **d**) 10 μM ATRA, **e**) 10 μM of EC23 and MEK1/2 inhibitor, **f**) SH-SY5Y cells with 10 μM EC23 (scale bar = 18 μm). Error bars indicate SEM of three biological replicates (** *p* < 0.01; *** *p* < 0.001; **** *p* < 0.0001 two-way ANOVA with Sidak’s multiple comparison test)

To confirm that non-genomic activity is necessary for retinoids to promote neurite outgrowth, kinase activity was blocked using the MEK1/2 inhibitor U0126 (Tocris) at a concentration of 10 μM. The MEK1/2 inhibitor U0126 produced a significant decrease in the neurite outgrowth induced by ATRA and EC23 (Fig. 7); neurite outgrowth was decreased 2.7-fold and 3.3-fold in ATRA- and EC23-treated cells, respectively, after adding the MEK1/2 inhibitor. MEK1/2 lies upstream of the ERK signaling pathway and these results imply that the MEK1/2 – ERK1/2 kinase signal transduction pathway is important in facilitating ATRA-induced neurite outgrowth. In addition, MEK1/2 inhibitor did not have an effect on SH-SY5Y cell number compared to control (results not shown).

## Discussion

Since the discovery of RA receptors as members of the nuclear receptor family of transcriptional regulators [49], the emphasis on retinoid function has been on their control of gene expression and a large number of synthetic retinoids have been generated [50] and investigated for their genomic activating properties. However, nuclear receptors can also have important non-genomic actions [7, 51]. Such non-genomic roles have also been proposed for the RA receptors, although the topic has remained relatively unexplored and is complex, with RA controlling multiple non-genomic pathways including regulation of kinases [52] and protein translation [53].

In this study, the 28 retinoids investigated varied in their genomic activity according to the differences in molecular structure and their relative affinities for the different receptors. For example, EC23 and GZ25, compounds that exhibit strong binding affinities for the RARs, were the most potent, followed by TTNPB, DC128, AH61, and DC271. Conversely, compounds with low affinity for the RARs, such as the RXR ligands DA124 and HX600 or the extended DC324 and DC329, did not induce a genomic response. In these respects, the results from the X-gal activity assays and the genomic activity of the compounds as inducers of *RARβ* and *CYP26A1* expression in SH-SY5Y cells were comparable, and the most potent ligands in the X-Gal reporter assay were also strong inducers of *RARβ* and *CYP26A1*. Nevertheless, in these experiments the *CYP26A1* gene was induced to a much higher extent than the *RARβ* gene. This could be because the *CYP26A1* gene has two RAREs that work synergistically [54]. Furthermore, the basal levels of *CYP26A1* expression were very low; hence, when the cells are treated with retinoids the fold increase in *CYP26A1* will be very high compared to the fold change in *RARβ*.

In the ERK1/2 phosphorylation assays, several retinoids lacked the ability to phosphorylate ERK1/2, whereas others were more effective than ATRA. Use of this assay helped us to identify novel properties for many of these compounds and little connection was found between induction of genomic versus non-genomic signalling, suggesting that quite different pathways mediate these two activities. TTNPB, DC318, DC444 and JBGG179, which were potent activators of gene transcription, did not show any detectable capacity to induce ERK1/2 phosphorylation compared to ATRA. In contrast, HX600, DA124, EC19, CD2665, DC375, DC324, DC329, DC476 and DC479, which did not have genomic activity, induced ERK1/2 phosphorylation. In addition, we show that the RAR β/γ antagonist CD2665 could act as an agonist for ERK 1/2 phosphorylation. Moreover, two ligands for the RXR class of RA receptors (HX600 and DA124) induced ERK 1/2 phosphorylation, revealing a novel property of this class of ligand.

**Fig. 8:**
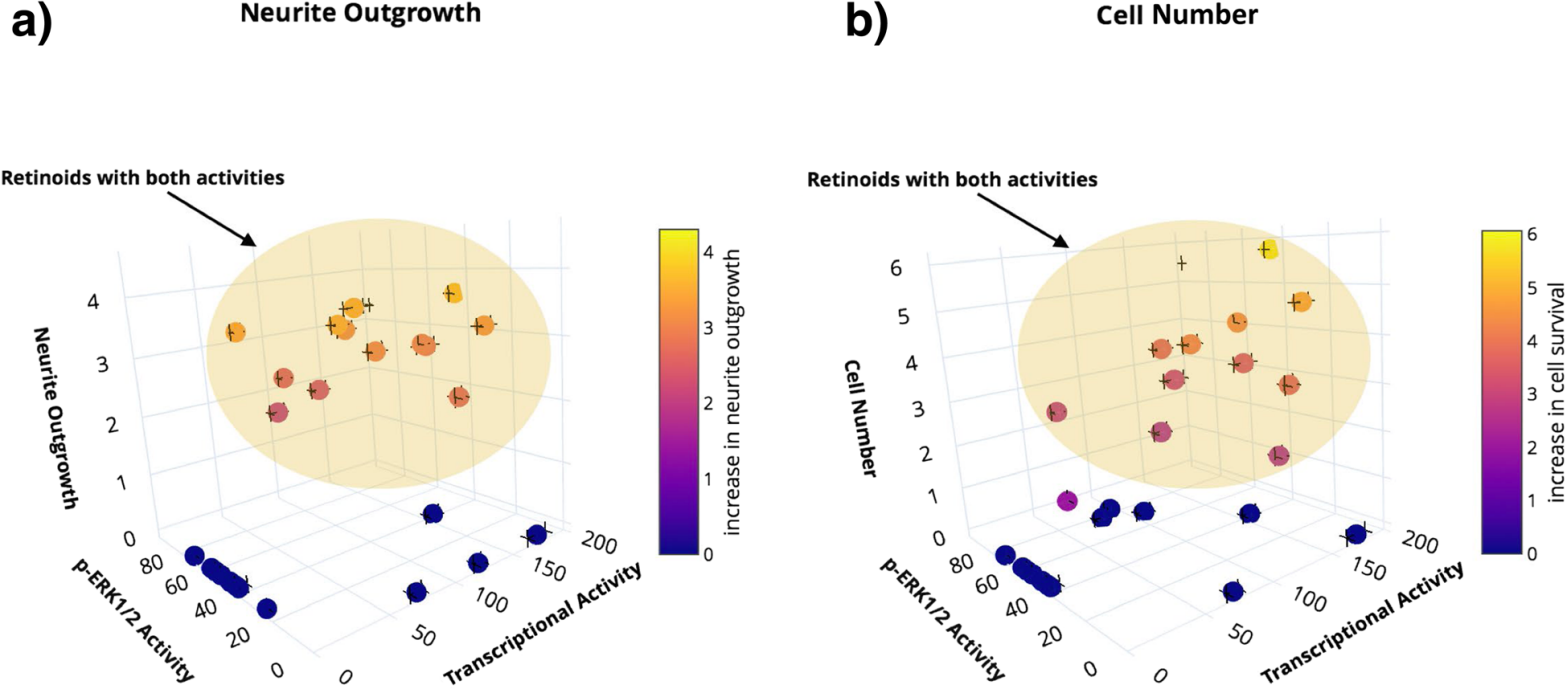
The relationship between the genomic and non-genomic activities and neurite outgrowth and cell number in SH-SY5Y cells. Retinoids with both genomic and non-genomic inducing activities had the highest enhancing effects on **a**) neurite outgrowth and **b**) cell number

While the activities of the retinoids in the separate genomic and non-genomic assays suggest that these functions are determined by different structural properties, a key result is that retinoids with *both* genomic and non-genomic inducing activities had the highest enhancing effects on neurite outgrowth, while compounds with only one activity tended to induce neurite outgrowth with lower efficiency (Fig. 8a). For example, EC23, AH61 and GZ25, which had the greatest enhancing effect on neurite length, were also strong inducers of gene transcription and ERK1/2 phosphorylation. Retinoids with only one activity did not induce neurite formation at low (10 nM) treatment concentrations.

When testing the correlation between retinoid transcriptional potency or ERK1/2 activation potency with neurite outgrowth or cell number, transcriptional activation activity correlated with neurite outgrowth and cell number while ERK1/2 potency correlated with neither. However, retinoids without detectable ERK1/2 induced significantly shorter neurites compared to retinoids with ERK1/2 inducing activity. Further, inhibitors of ERK1/2 activity blocked retinoid induced neurite outgrowth. These data indicate that ERK1/2 is required for retinoid-induced neurite outgrowth and influences length but it is potency of transcriptional activity of the retinoids that is directly proportional to neurite outgrowth. The induction of ERK1/2 activity by retinoids did not appear to influence cell number, although one mechanism by which retinoids increase number of SH-SY5Y cells is through increased cell survival in an ERK1/2 dependent mechanism [9]. Because the time scales of the ERK1/2 and transcriptional assays differ by an order of magnitude, and the neurite and cell-number responses are measured after 5 days, it would be premature to discount a continuing role of ERK1/2 in control of cell number. To address this issue adequately, time-course analyses guided by computational modelling, coupled with bioinformatic approaches to data collection and analysis, will be required.

One mechanism by which ATRA promotes neurite outgrowth is through regulation of the transcription of neurotrophin genes, such as nerve growth factor (NGF) and brain derived neurotrophic factor (BDNF), which are important regulators of survival and neurite outgrowth [55, 56], and increasing the expression of TrkB, the receptor for BDNF in SH-SY5Y cells [17, 45]. Furthermore, a number of genes that are downstream targets of ATRA genomic activity have been shown to function as neurite regulating factors [57–61].

A second mechanism of ATRA induced neurite outgrowth is through kinase activation. A number of kinase pathways have been associated with ATRA’s ability to induce neurite outgrowth in neuroblastoma cells such as ERK1/2 [62, 63], c-Jun *N*-terminal kinase (JNK) [64–66] and PI3K [67]. The action in this study of the MEK1/2 inhibitor to reduce neurite outgrowth in EC23 and ATRA treated cells supports the importance of the MEK1/2-ERK1/2 pathway. These different kinase pathways have been shown to play an important role in cytoskeletal reorganization and the modulation of neurite outgrowth [56, 68]. For example, phosphorylated ERK1/2 regulates microtubule dynamics by phosphorylating different microtubule associated proteins and it also enters the nucleus and activates various transcription factors such as cAMP response element binding (CREB) protein required for axonal growth [56]. Yu and colleagues [66] reported that the JNK pathway plays a role in ATRA induced neurite outgrowth in SH-SY5Y cells as neurite outgrowth in the cells was repressed after inhibiting JNK. Lee and Kim [64] showed that the ERK pathway is essential for ATRA induced neurite outgrowth in SK-N-BE(2)C neuroblastoma cells. Additionally, it was reported that ATRA induced PI3K leads to the activation of the Rac1 G-protein, which is important for neuritogenesis and PI3K inhibition impaired ATRA induced neurite outgrowth in SH-SY5Y cells [67].

The routes by which the retinoids described in this study regulate non-genomic actions are very diverse, frequently rapid and involve a receptor in the cytoplasm. RARα is particularly prominent in such a role. In the cytoplasm RARα acts as an mRNA binding protein to regulate protein translation [53]. It is also present in lipid rafts of the plasma membrane (in MCF7 cells), and retinoic acid rapidly activated P38MAPK in MCF7, HeLa cells, F9 mouse embryocarcinoma cells and mouse embryonic fibroblasts with Gq protein α subunit acting as an intermediate [69]. Alternatively, in SH-SY5Y cells RARα, again at the plasma membrane, interacts with PI3K to activate ERK1/2 [70] while in the cytoplasm of neuronal LA-N-5 cells RARγ interacts with c-src kinase to control neurite outgrowth [71]. The complexity of pathways does not end there. The cytoplasmic receptor for RA and related compounds mediating non-genomic actions do not necessarily need to be an RAR. For instance, a cytoplasmic function for RA in CJ7 embryonic stem cells and COS1 (kidney) cells to induce rapid ERK1/2 activation uses cellular retinoic acid binding protein 1 (CRABPI) [72]. RA can itself directly bind to kinases such as Protein kinase C alpha [73] while in the neuronal cell line PC12 CaMKII has a vital role to play in RA activation of ERK1/2 [74]. Thus, some of the actions of the compounds described in the study to activate ERK1/2 may not be through the RARs themselves.

## Conclusions

This study showed that the tested retinoids varied in their ability to activate gene transcription and the non-genomic pathways via ERK1/2 activation, and that some compounds exhibit activity corresponding to the modulation of only a single pathway. Furthermore, ligands that activate both genomic and non-genomic pathways had the highest activity in assays for neuronal cell number and neurite outgrowth. Thus, such dual-potency retinoids can be developed in order to considerably enhance their activity as promoters of neurite outgrowth and neuronal cell number. Alternatively, retinoids with pathway specificity can be developed to potentially reduce side-effects. These results have important implication for the further development of these drugs for neurodegenerative disorders.

## Additional files

Additional file 1: **Table S1.** Summary of effects of retinoids on the genomic, non-genomic and neurite outgrowth activities in SH-SY5Y cells. The EC_50_ value represents the half-maximal effective concentration and is related to the affinity for the RA receptor, while the maximum stimulation, which is defined by the E_max_ value, is a measure of compound efficacy. The 95% confidence intervals (CI) represents the predicted range of EC_50_ and E_max_ values for a specific compound. Fold increase represents the increase in neurite formation compared to non-treated cells. (PDF 73 kb)

Additional file 2: **Figure S1.** Correlation analyses between the effects of retinoids (filled circles) on cell number, neurite length, transcriptional activity (Efficacy, E_max_) and ERK1/2 activity (Efficacy, E_max_). Lines (non-parametric median slopes) are only fitted where correlation coefficients (Pearson, or Spearman for ranked plots), as given in the main text, were significant (*P* < 0.05). a, cell number versus neurite length (fold change); b, ranked ERK1/2 activity versus ranked transcriptional activity for all retinoids; c, neurite length (fold change) versus ERK1/2 activity for retinoids that induced an increase in neurite length; d, ranked neurite length (fold change) versus ranked transcriptional activity for retinoids that induced an increase in neurite length; e, cell number (fold change) versus ERK1/2 activity for all retinoids; f, ranked cell number (fold change) versus ranked transcriptional activity for all retinoids. (DOCX 123 kb)

## Abbreviations

ATRA: All-*trans*-retinoic acid
BDNF: Brain derived neurotrophic factor
C_6_FeK_3_N_6_: Potassium ferricyanide
C_6_FeK_4_N_6_: Potassium ferrocyanide
cDNA: Complement deoxyribonucleic acid
CI: Confidence intervals
CT: Control, Untreated cells
CYP26: Cytochrome P450, family 26
DMEM: Dulbecco’s Modified Minimum Essential Medium
DMSO: Dimethyl sulfoxide
DNA: Deoxyribonucleic acid
EC_50_: Half maximal effective concentration
EDTA: Ethylenediaminetetraacetic acid
E_max_: Maximum possible effect for the agonist
ERK: Extracellular signal-regulated kinases
FCS: Fetal calf serum
g: Gram(s)
GAPDH: Glyceraldehyde 3-phosphate dehydrogenase
GIMP: GNU image manipulation program
h: Hour(s)
HCl: Hydrochloric acid
HNO3: Nitric acid
JNK: c-Jun N-terminal kinase
M: Molar
MEK1/2: Mitogen-activated protein kinase kinase family member
mg: Milligram(s)
MgCl_2_: Magnesium chloride
min: Minute(s)
ml: Millilitre(s)
mm: Millimetre(s)
mM: Millimolar
mRNA: Messenger ribonucleic acid
ng: Nanogram(s)
NGF: Nerve growth factor
nm: Nanometre(s)
nM: Nanomolar
PBS: Phosphate-buffered saline
PBST: Phosphate-buffered saline with 0.1% triton X-100
PCR: Polymerase chain reaction
p-ERK: Phosphorylated ERK
PFA: Paraformaldehyde
PI3K: Phosphoinositide 3- kinase
PLL: Poly-L-lysine
qPCR: Quantitative polymerase chain reaction
RA: Retinoic acid
RAR: Retinoic acid receptor
RARE: Retinoic acid response element
RBP4: Retinol binding protein 4
RNA: Ribonucleic acid
RT: Reverse transcriptase
RXR: Retinoic X receptor
s: Second (s)
SEM: Standard error of the mean
X-Gal solution: 5-Bromo-4-Chloro-3-Indolyl β-D-Galactopyranoside
μg: Microgram(s)
μl: Microliter(s)
μM: Micromolar
μm: Micron or Micrometre(s)
