# Supplementary material for: Genomic and non-genomic pathways are both crucial for peak induction of neurite outgrowth by retinoids
Source: Cell Commun Signal. 2019 May 2;17:40. doi: 10.1186/s12964-019-0352-4 (PMC6498645; doi:10.1186/s12964-019-0352-4)
Supplement: Supplementary file 1 — Table S1. Summary of effects of retinoids on the genomic, non-genomic and neurite outgrowth activities in SH-SY5Y cells. The EC50 value represents the half-maximal effective concentration and is related to the affinity for the RA receptor, while the maximum stimulation, which is defined by the Emax value, is a measure of compound efficacy. The 95% confidence intervals (CI) represents the predicted range of EC50 and Emax values for a specific compound. Fold increase represents the increase in neurite formation compared to non-treated cells. (PDF 73 kb) [file 12964_2019_352_MOESM1_ESM.pdf]

Compound	Genomic Activity (X-Gal RA Based Reporter Assay)		Non-Genomic Activity (ERK1/2 Phosphorylation Assay)		Neurite Outgrowth	
	Potency (EC_{50}) "nM" (95% CI)	Efficacy (E_{max}) (95% CI)	Potency (EC_{50}) "nM" (95% CI)	Efficacy (E_{max}) (95% CI)	Fold increase (10 µM)	Fold increase (10 nM)
ATRA	1.044 (0.8396 to 1.291)	170.1 (164.4 to 175.7)	33.07 (23.76 to 44.85)	48.55 (46.72 to 50.42)	2.8	Did not induce neurite outgrowth
AH61	1.954 (1.361 to 2.821)	196.1 (185.1 to 207.4)	18.07 (9.765 to 35.5)	44.46 (40.82 to 48.35)	3.6	1.9
EC23	0.1279 (0.08006 to 0.2033)	169.18 (159.1 to 180.7)	30.55 (19.47 to 48.53)	92.51 (85.7 to 99.62)	4.3	2.2
EC19	Did not induce a genomic response		50.71 (14.77 to 118)	22.65 (19.69 to 25.62)	Did not induce neurite outgrowth	
TTNPB	0.09958 (0.06777 to 0.1462)	108.5 (102.8 to 114.2)	Did not induce a non-genomic response		2.5	Did not induce neurite outgrowth
Fenretinide	52.78 (36.88 to 74.29)	150.9 (138.8 to 163.7)	90.8 (58.85 to 138.4)	36.87 (34.15 to 39.68)	Killed the cells	1.7
A1120	107.1 (69.46 to 163.2)	93.76 (83.61 to 105)	4.323 (2.302 to 7.727)	44.87 (41.83 to 47.98)	3.4	1.7
CD2665	Did not induce a genomic response		4.849 (2.969 to 7.672)	44.3 (41.6 to 47.05)	Did not induce neurite outgrowth	
DA124	Did not induce a genomic response		18.07 (9.765 to 35.5)	44.46 (40.82 to 48.35)	Did not induce neurite outgrowth	
HX600	Did not induce a genomic response		14.22 (9.415 to 21.28)	69.15 (65.02 to 73.35)	Did not induce neurite outgrowth	
GZ25	0.1838 (0.1385 to 0.245)	209.1 (201.4 to 216.9)	3.57 (2.835 to 4.484)	76.62 (74.03 to 79.24)	3.3	1.9
DC122	24.56 (14.56 to 41.67)	89.48 (80.75 to 99.01)	143.5 (117.8 to 174.9)	77.63 (74.75 to 80.58)	2.4	Did not induce neurite outgrowth
DC128	1.399 (0.8395 to 2.384)	192 (177.9 to 206.3)	30.37 (21.26 to 43.1)	28.85 (27.3 to 30.44)	3.1	1.7
DC271	2.284 (0.8761 to 5.796)	157.1 (136.4 to 179.6)	111 (76.63 to 159.1)	78.37 (73.54 to 83.36)	3.4	1.7
DC303	7.98 (4.335 to 13.73)	103.2 (90.96 to 115.9)	390.2 (350.4 to 434.2)	65.98 (64.42 to 67.57)	2.2	Did not induce neurite outgrowth
DC318	24.9 (31 to 48.73)	69.38 (60.4 to 79.9)	Did not induce a non-genomic response		Did not induce neurite outgrowth	
DC324	Did not induce a genomic response		26.35 (19.51 to 35.49)	50.22 (47.4 to 53.09)	Did not induce neurite outgrowth	
DC329	Did not induce a genomic response		15.66 (12.25 to 20.1)	40.68 (39.24 to 42.15)	Did not induce neurite outgrowth	
DC360	18.85 (8.9 to 39.51)	58.1 (50.07 to 66.89)	159.4 (117.7 to 215.5)	79.73 (75.14 to 84.46)	3.3	Did not induce neurite outgrowth
DC375	Did not induce a genomic response		2726 (1701 to 4774)	38.84 (33.67 to 46.62)	2.8	Did not induce neurite outgrowth
DC440	118.8 (84.33 to 165.1)	153 (138 to 169.3)	9.313 (5.603 to 15.31)	82.28 (77.06 to 87.62)	3	Did not induce neurite outgrowth
DC444	6.97 (3.599 to 12.93)	172.4 (154.4 to 191.2)	Did not induce a non-genomic response		3.1	Did not induce neurite outgrowth
DC472	32.7 (14.52 to 71.19)	76.12 (63.96 to 90.78)	3.496 (2.147 to 5.656)	72.45 (68.39 to 76.62)	1.9	Did not induce neurite outgrowth
DC476	Did not induce a genomic response		2.004 (1.39 to 2.93)	53.91 (51.5 to 56.36)	1.9	Did not induce neurite outgrowth
DC479	Did not induce a genomic response		501.6 (260.3 to 854.2)	56.77 (50.45 to 63.47)	Did not induce neurite outgrowth	
TTNN	0.8526 (0.373 to 1.784)	118.6 (105.8 to 131.7)	2.491 (1.966 to 3.152)	42.44 (40.92 to 43.98)	2.9	1.5
JBGG179	6.411 (4.612 to 8.728)	118.5 (111.9 to 125.3)	Did not induce a non-genomic response		2.2	Did not induce neurite outgrowth
EC23Al	64.31 (32.67 to 114.9)	210.2 (181.9 to 241.2)	6.349 (46.62 to 84.46)	94 (89.63 to 98.43)	3.3	Did not induce neurite outgrowth
DC547	29.79 (17.85 to 49.54)	181.9 (163.9 to 201.4)	484.4 (353.4 to 654.5)	53.07 (49.48 to 56.83)	2.7	Did not induce neurite outgrowth
