# Supplementary material for: Genomic and non-genomic pathways are both crucial for peak induction of neurite outgrowth by retinoids
Source: Cell Commun Signal. 2019 May 2;17:40. doi: 10.1186/s12964-019-0352-4 (PMC6498645; doi:10.1186/s12964-019-0352-4)
Supplement: Supplementary file 2 [file 12964_2019_352_MOESM2_ESM.docx]

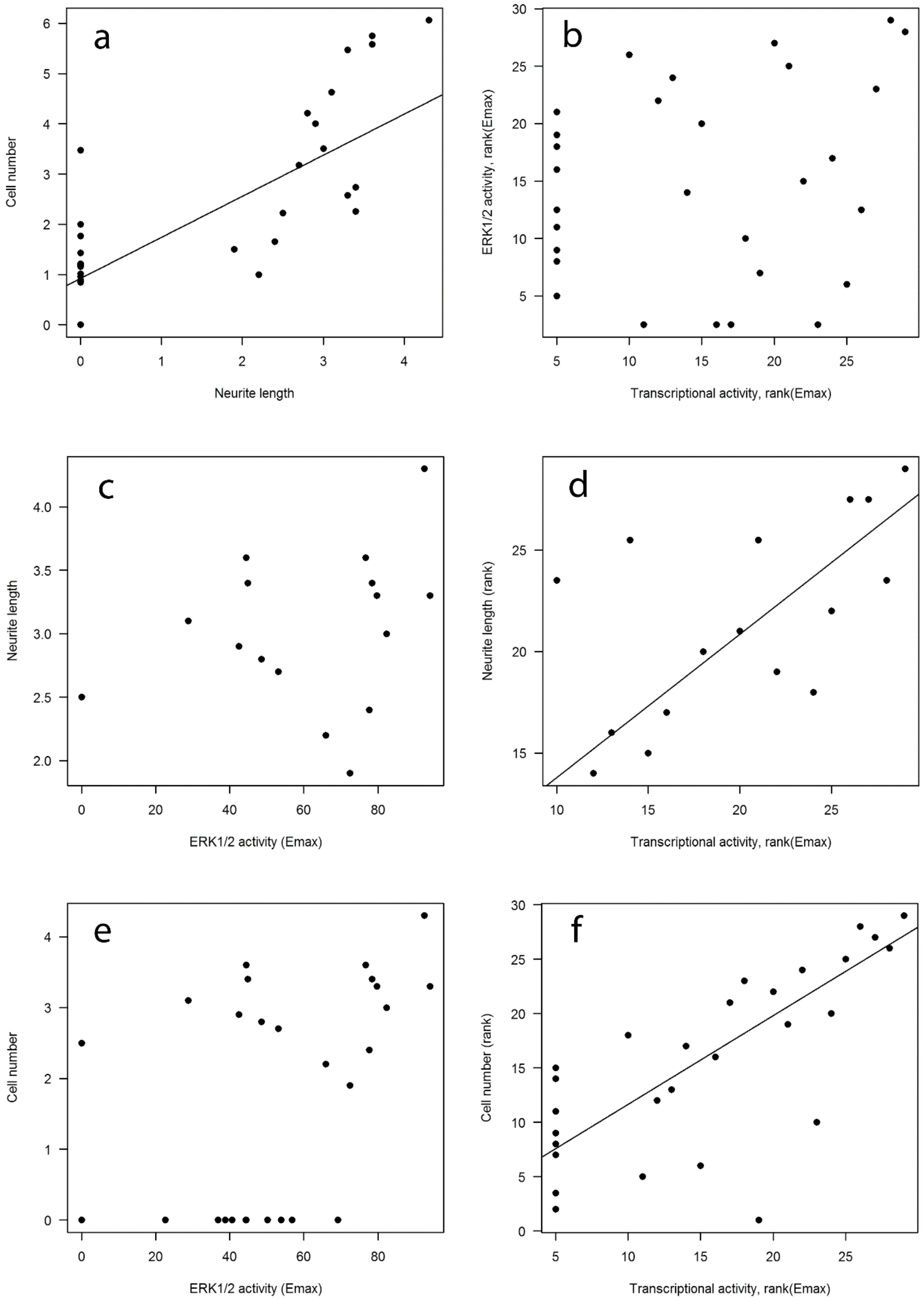

a
Cell number
Neurite length
b
ERK1/2 activity, rank(Emax)
Transcriptional activity, rank(Emax)
c
Neurite length
ERK1/2 activity (Emax)
d
Neurite length (rank)
Transcriptional activity, rank(Emax)
e
Cell number
ERK1/2 activity (Emax)
f
Cell number (rank)
Transcriptional activity, rank(Emax)
